# HPV-16 infection modifies overall survival of Puerto Rican HNSCC patients

**DOI:** 10.1186/s13027-016-0095-4

**Published:** 2016-08-24

**Authors:** Bianca Rivera-Peña, Francisco J. Ruíz-Fullana, Germán L. Vélez-Reyes, Rosa J. Rodriguez-Benitez, María J. Marcos-Martínez, Juan Trinidad-Pinedo, Adriana Báez

**Affiliations:** 1 Department of Biology, Natural Sciences Faculty, University of Puerto Rico, Rio Piedras Campus, San Juan, Puerto Rico; 2 Department of Otolaryngology, Head and Neck Surgery Section, University of Puerto Rico, School of Medicine, PO Box 365067, San Juan, 00936 Puerto Rico; 3 Department of General Social Sciences, University of Puerto Rico, Rio Piedras Campus, San Juan, Puerto Rico; 4 Department of Pathology and Laboratory Medicine, University of Puerto Rico, School of Medicine, San Juan, Puerto Rico; 5 Medical Services Administration, San Juan, Puerto Rico; 6 Department of Pharmacology and Toxicology, University of Puerto Rico, School of Medicine, San Juan, Puerto Rico

**Keywords:** Head and neck squamous cell carcinoma, Human Papillomavirus (HPV), Overall Survival (OS), TNM, Biomarker

## Abstract

**Background:**

HPV-16 modifies the overall survival (OS) of patients with oropharyngeal cancer (OPSCC). HPV-16 has been established as risk factor for OPSCC, but HPV-16 infection may also reside in the larynx and oral cavity. We evaluated HPV-16 status on OS of Head and Neck Squamous Cell Carcinoma (HNSCC) patients.

**Methods:**

HPV-16 infection was confirmed by amplification of E6 and E7 viral oncogenes through PCR assay and E6 IHC in 185 HNSCC samples. Associations between HPV-16 status and clinicopathological parameters were performed using Fisher’s exact test and x^2^. Survival analysis was completed using Kaplan-Meier estimator and multivariate Cox regression analysis.

**Results:**

OS of HPV-16 positive patients was longer compared to HPV-16 negative patients (*P* = 0.002). HPV-16 positive tumors of the larynx (LSCC) and pharynx (PSCC) showed improved OS compared to HPV-16 negative tumors. Also, HPV-16 positive patients exposed to radiotherapy presented a better survival.

**Conclusions:**

HPV-16 status has a positive prognostic value in HNSCC. Addition of HPV-16 status to the TNM staging can provide better assessment in prognosis and guide treatment for HNSCC patients.

## Background

HNSCC is the seventh most common type of cancer diagnosed, and it is ranked as the eighth cause of cancer death worldwide [1, 2]. This cancer includes tumors from the oral cavity (OSCC) (ICD-10-C14.8), pharynx (PSCC) (ICD-10-C14.0), larynx (LSCC) (ICD-10-C32.9), and the paranasal sinuses (ICD-10-C31.9) [3]. HNSCC is predominantly diagnosed in patients over 60 years old; however, a growing number of HNSCC patients are being diagnosed at younger ages [4]. Historically, HNSCC has been more frequently diagnosed in men, with a male–female ratio of about 4:1. However, this ratio is rapidly changing because more women are exposing themselves to tobacco and alcohol [5]. The overall-5-year survival (OS) for HNSCC patients is 65.9 %, for all HNSCC sites and stages [6], with a median survival of 2.5 years after treatment.

In the United States, Puerto Rican Hispanics, African-Americans, and economically disadvantaged Whites are at greater risk of developing HNSCC. The incidence of HNSCC in Puerto Ricans is 2.5 higher than Hispanics in the US [7]. The incidence of OSCC or PSCC is approximately 72 % higher in Puerto Ricans than in US Hispanics. Similarly, the incidence rate of LSCC is 51 % higher than among Hispanics living in the Unites States [7].

The etiology of HNSCC involves a variety of toxic, environmental, and viral agents [5]. Studies have established that smoking and alcohol consumption are the major risk factors for the development of HNSCC [8–10]. Currently, human papillomavirus (HPV) infection has also been recognized as a risk factor for HNSCC, particularly for OPSCC [11–13]. There are more than 180 types of HPVs described, of which 30 types are considered high risk, including HPV-16 and HPV-18 [14, 15]. The malignant transformation of HPV integration is mediated by HPV oncoproteins E6 and E7 [14]. HPV-16 E6 protein has been associated to the abnormal degradation of the p53 protein, leading to a disruption in G1/S cell cycle control [16]. Also, HPV-16 E7 oncoprotein binds to the phosphorylated form of pRb protein, which inactivates pRb and a disruption in the G1/S transition occurs [17]. Both events cause an abnormal promotion of cell proliferation due to disruptions in the cell cycle control mechanisms. HPV-16 DNA has been detected in almost 35 % of HNSCC patients, and evidence has accumulated showing that HPV is etiologic for OPSCC [18, 19]. It has been proposed that HPV-16 positive HNSCC patients have a distinct cancer progression and prognosis than HPV-16 negative HNSCC patients [20]. HPV-16 positive patients tend to be diagnosed at a younger age when compared to HPV-16 negative patients [20, 21]. Additionally, the presence of HPV-16 in HNSCC patients has been correlated to the presence of local metastases, positive lymph nodes, and a more advanced tumor stage at the time of diagnosis [22]. Clinically, HPV-16 positive HNSCC patients have a better prognosis than HPV-16 negative patients [11, 13, 23, 24].

The complex anatomical structure of the head and neck area makes it very challenging for clinicians to determine the primary site of HNSCC [25]. Detection of HNSCC involves clinical and histological examinations of suspicious tissue, but, at times, unnoticed malignant lesions remain undetected. HNSCC tumors arising from each anatomical site have a unique progression, epidemiology, and therapeutic approach. HNSCC prognostication is based on the TNM Classification of Malignant Tumors (TNM) according to the sub-site [26]. The TNM system is useful to describe the extent of the disease, estimate the likely prognosis, and plan treatment. Treatment strategies rely on TNM, possible side effects, and the patient’s preferences and overall health. Since HNSCC is often discovered in advance stages (III and IV), the most urgent problem is the need to identify an effective diagnostic marker for early detection, and prediction of outcome. Therefore, the purpose of this study was to evaluate whether addition of HPV-16 status to the TNM staging system will help predict better the OS of HNSCC Puerto Rican patients.

## Methods

### Study design

This is a retrospective study where patients meeting the following criteria were eligible for inclusion: histologically proven squamous cell carcinoma arising from the pharynx (hypopharynx, oropharynx), oral cavity, and larynx treated surgically between 1993 and 2005. Fresh-frozen tumor tissue was collected from all HNSCC accrued patients. Additionally, genomic DNA of HNSCC patients had been previously tested for HPV-16 status by Gp5+/6+ primer region within the L1 gene consensus PCR [27], HPV-16 E6/E7 type-specific PCR, and E6 immunohistochemical (IHC) staining [13, 28, 29]. The cohort consisted of 185 HNSCC and their clinicopathological parameters are shown in Table 1. All procedures have the approval of the University of Puerto Rico-Medical Sciences Campus IRB (MSC-IRB Protocol 2770103). Relevant diagnostic information including tumor site, tumor grade, and histology were obtained from medical records and pathological reports. Treatment of choice was surgery followed by postoperative radiotherapy. Follow-up information was prospectively collected from hospital, pathological records and the Puerto Rican Cancer Registry.

**Table 1:** Study cohort clinicopathological characteristics

Characteristics	*N* = 185
Age (y)	
Mean ± SD	62.72 ± 12.13
Range	24-98
Sex, n (%)	
Male	164 (88.6)
Female	21 (11.4)
Primary Tumor Site, n (%)	
Larynx	83 (44.9)
Oral Cavity	68 (36.7)
Oropharynx	17 (9.2)
Hypopharynx	17 (9.2)
HPV-16 Status, n (%)*	
HPV-16 +	97 (52.4)
HPV-16 -	88 (47.6)
Tumor Stage, n (%)	
I, II	47 (25.4)
III, IV	138 (74.6)
Tumor Grade, n (%)	
Well	43 (23.2)
Moderate	107 (57.8)
Poor	16 (8.7)
SCC	19 (10.3)
Nodal Involvement	
Yes	64 (34.6)
No	121 (65.4)
Heavy Smoking, n (%)	163 (88.1)
Heavy Drinking, n (%)	154 (83.2)

*HPV-16 + = human papillomavirus type 16 positive; HPV-16 - = human papillomavirus type 16 negative

### DNA extraction

Genomic DNA from all tumor samples was isolated using the DNA Isolation kit for Cells and Tissues (Roche, Indianapolis, IN) according to the manufacturer instructions. DNA concentration was measured with NanoDrop 8000 UV–vis Spectrophotometer (Thermo Scientific, Waltham, MA).

### Detection of HPV16 DNA

HPV-16 status had been pre-screened by Gp5+/6+ consensus PCR followed by HPV-16 E6/E7 type-specific PCR, and results were confirmed for this study with a TaqMan-based qPCR targeted at HPV-16 E6 and E7 viral oncogenes. The HPV-16 E6 specific primer set included a forward primer 5′-gcacagagctgcaaacaactataca-3′, a reverse primer 5′-tcccgaaaagcaaagtcatatacc-3′, and a probe oligo 5′-tgtactgcaagcaacagttactgcgacgt-3′. The HPV-16 E7 specific primer set included a forward primer 5′-gatgaaatagatggtccagc-3′, a reverse primer 5′-gctttgtacgcaaccgaagc-3′, and a probe oligo 5′-cggacagagcccattacaatattgtaacc-3′. Quality and amount of input DNA samples were tested in each qPCR assay with *β-actin* gene primers with a forward primer 5′-gcccatctacgaggggta-3′, a reverse primer 5′-ccttaatgtcacgcacga-3′, and a probe oligo 5′-accaccacggccgagcgg-3′. Reaction mixtures with SiHa DNA (1–2 copies of HPV-16) and K562 DNA (HPV-16 negative) were used as positive and negative control, respectively. qPCR reactions were carried out in a 96-well optical tray with a final volume of 25 μL. Each reaction consisted of 600 nM of each primer, 200 nM of each probe (Taqman, Applied Biosystems, Grand Island, NY), 1X of TaqMan Universal PCR Master Mix (Applied Biosystems), which contains the Taq Polymerase, dNTPs, and ROX reference dye, and 75 ng of genomic DNA. DNA was amplified in a 7500 Real Time PCR System (Applied Biosystems, Grand Island, NY). Thermal cycling conditions were: 50 °C for 2 min, 95 °C for 10 min, followed by 40 cycles of 95 °C for 15 s and an annealing temperature of 60 °C for 1 min. All HNSCC samples classified as HPV-16 positive had amplification of E6 and E7 viral oncogenes through qPCR assay.

### Statistical analysis

Data from independent groups was compared using Fisher exact test or x^2^, as appropriate. Odds ratio (OR) calculations for clinicopathological parameters were performed using binary logistic. Overall survival (OS) was measured in months from the date of diagnosis until death, if occurred. Survival analyses were performed using Kaplan-Meier curves. Log-rank Mantel-Cox and Gehan-Breslow Wilcoxon tests were used to determine the significance between two survival curves. Established prognostic factors having an impact on HNSCC survival were analyzed in a multivariate Cox regression analysis. Statistical significance was established to be *p* ≤ 0.05. Statistical analyses were performed using IBM SPSS Version 22 (IBM Corp; Armonk, NY).

## Results

### HPV-16 status in a cohort of HNSCC Puerto Rican patients

Patient characteristics are shown in Table 1. The mean age was 62.72 years (range 24–98 SD: 12.13). Of the 185 HNSCC patients included in the study, 88.6 % were male and 11.4 % were female. Three-quarters (74.6 %) of the patients presented tumors with advance staging (III and IV) and 25.4 % were in early stages (I and II) of the disease. The HNSCC sub-site distribution was 44.9 % LSCC, 36.7 % OSCC and 18.4 % PSCC. The PSCC sub-site includes cases from the oropharynx and hypopharynx. Patients with oropharyngeal and hypopharyngeal cancers were combined under PSCC for the statistical analysis in view of the relatively small number of cases of each sub-site in our study cohort. However, the distribution of oropharyngeal and hypopharyngeal cancers is shown in Table 1. Smoking and drinking habits of our study cohort were defined according to the substance usage reported by each consented patient. Heavy smoking patients reported smoking a pack or more of cigarettes per day and heavy drinking patients reported 15 drinks or more per week. The majority of our HNSCC patients were heavy smokers (88.1 %) and heavy drinkers (83.2 %).

When we distribute the study cohort by HPV-16 status, 52.4 % were HPV-16 positive and 47.6 % were HPV-16 negative. The HNSCC sub-site distribution of the HPV-16 positive patients was 42.3 % LSCC, 37.1 % were OSCC and 20.6 % were PSCC. No statistically significant association was found between HPV-16 status and gender, age, risk factors and staging (Table 2).

**Table 2:** Adjusted OR’s and 95 % CIs of HNSCC patients according to HPV-16 status and clinicopathological parameters

Variable	HPV-16 + (*N* = 97)		HPV-16 - (*N* = 88)		OR value, 95 % CI	*P* value
	No.	Percent	No.	Percent		
Age (y)						
≤ 60	45	46.4	38	43.2		
> 60	52	53.6	50	56.8	1.14 [0.637 – 2.035]	0.767
Sex						
Male	89	91.8	75	85.2		
Female	8	8.2	13	14.8	1.93 [0.759 – 4.902]	0.173
Tumor site						
Larynx	41	42.3	42	47.7	0.866 [0.304 – 2.470]	0.788
Oral Cavity	36	37.1	32	36.4	0.998 [0.338 – 2.942]	0.996
Pharynx	20	20.6	14	15.9	1.675 [0.420 – 6.681]	0.465
Stage of Disease						
I,II	30	30.9	17	19.3		
III,IV	67	69.1	71	80.7	1.87 [0.945 – 3.700]	0.091
Tumor grade						
SCC	11	11.3	8	9.1		
Well	22	22.7	21	23.9	0.875 [0.226 – 3.385]	0.847
Moderate	55	56.7	52	59.1	0.729 [0.269 – 1.973]	0.534
Poor	9	9.3	7	7.9	0.720 [0.240 – 2.161]	0.558
Nodal involvement						
Yes	30	16.2	34	18.4		
No	67	36.2	54	29.2	0.711 [0.387-1.306]	0.272
Tobacco status						
Yes	88	90.7	75	85.2		
No	9	9.3	13	14.8	1.70 [0.686 – 4.186]	0.265
Alcohol status						
Yes	82	84.5	72	81.8		
No	15	15.5	16	18.2	1.22 [0.561 – 2.630]	0.695

*HPV-16 +* human papillomavirus type 16 positive, *HPV-16 -* human papillomavirus type 16 negative, *OR* odds ratio, *95 % CI* 95 % confidence interval

### Survival analysis of HNSCC patients according to HPV-16 status

**Fig. 1:**
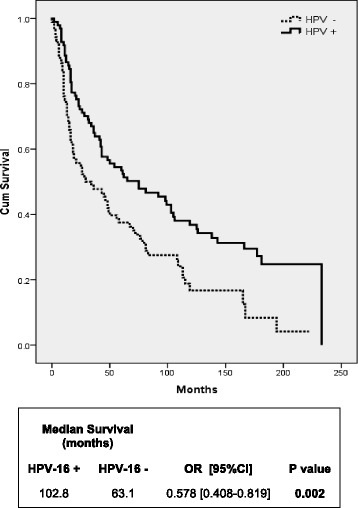
Overall survival of HNSCC patients according to HPV-16 status. Kaplan-Meier survival curve analysis of HNSCC patients according to HPV-16 status. Analysis includes all 185 HNSCC patients for all anatomical sub-sites and stages

HNSCC patients with HPV-16 positive status had a better OS compared to HPV-16 negative patients for all sites and stages (Fig. 1). HPV-16 positive patients had a median survival of 102.8 months compared to HPV-16 negative tumors, which had a 63.1 months median survival (*P* = 0.002). Cox regression analysis was performed and showed that HPV-16 positive HNSCC patients have an improved survival compared to HPV-16 negative patients after adjustment for established prognostic factors such as nodal status, tumor stage, cell differentiation, tumor site, heavy smoking and heavy drinking usage, age, and sex (Table 3).

**Table 3:** Multivariate Cox analysis

Covariate	Coefficient	Standard error	*P* value	HR value, 95 % CI
Positive nodal status	0.308	0.209	0.141	1.361 [0.903 – 2.052]
Tumor stage	0.034	0.099	0.733	1.034 [0.852 – 1.257]
Tumor grade				
Well	0.089	0.442	0.841	1.093 [0.460 – 2.598]
Moderate	0.348	0.322	0.279	1.417 [0.754 – 2.662]
Poor	0.496	0.344	0.149	1.642 [0.837 – 3.222]
Tumor site				
Larynx	- 0.170	0.337	0.614	0.844 [0.436 – 1.634]
Oral Cavity	- 0.176	0.350	0.616	0.839 [0.423 – 1.665]
Pharynx	0.237	0.423	0.575	1.267 [0.554 – 2.901]
Heavy smoking	0.202	0.329	0.539	1.224 [0.642 – 2.333]
Heavy drinking	0.217	0.249	0.384	1.242 [0.762 – 2.024]
HPV-16	0.555	0.182	0.002	1.742 [1.219 – 2.488]
Sex	- 0.247	0.324	0.447	0.781 [0.414 – 1.475]
Age	0.026	0.007	0.000	1.026 [1.011 – 1.041]

(−2 Log Likelihood): 1051.086, *HR* hazard ratio, *95 % CI* 95 % confidence interval, *HPV-16* human papillomavirus type 16

**Fig. 2:**
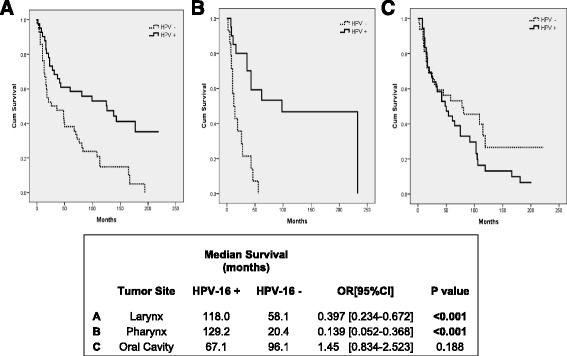
Overall survival of HNSCC patients according to HPV-16 status in each HNSCC anatomical sub-sites. Kaplan-Meier survival curve analysis of HPV-16 positive patients versus HPV-16 negative patients according to HNSCC anatomical sub-site. **a** Larynx (LSCC); **b** Pharynx (PSCC); **c** Oral Cavity (OSCC)

LSCC and PSCC patients with HPV-16 positive tumors had a better OS compared to HPV-16 negative patients (Fig. 2a, 2b). The median survival of HPV-16 positive LSCC patients was 118 months compared to HPV-16 negative cases which had a median survival of 58.1 months (*P* = <0.001). As well, the OS of PSCC patients improves in HPV-16 positive cases (129.2 months) compared to HPV-16 negative cases (20.4 months) (*P* = <0.001). In contrast, the OS of OSCC patients was not significantly different between HPV-16 positive and HPV-16 negative cases (Fig. 2c).

**Fig. 3:**
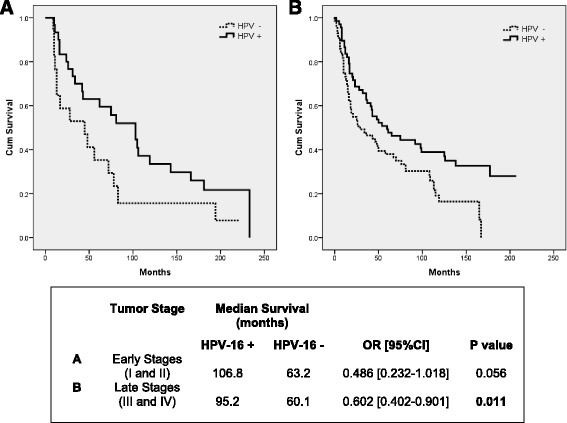
Overall survival of HNSCC patients according to HPV-16 status and tumor staging. Kaplan-Meier survival curve analysis of HNSCC HPV-16 positive patients versus HPV-16 negative patients distributed by tumor staging. **a** Early Stages (I and II); **b** Late Stages (III and IV)

Since HPV-16 positive patients showed a better survival, we evaluated the effect of HPV-16 presence on OS in HNSCC tumor staging. As shown in Fig. 3, HPV-16 positive patients have a better OS than HPV-16 negative patients regardless of the tumor staging. HPV-16 positive early stage tumors had an improved OS of 106.8 months as compared to HPV-16 negative tumors, which had a median OS of 63.2 months (*P* = 0.056) (Fig. 3a). Likewise, late stage tumors that were HPV-16 positive had a better OS (95.2 months) compared to late stages HPV-16 negative tumors, whose median OS was 60.1 months (*P* = 0.011) (Fig. 3b).

**Fig. 4:**
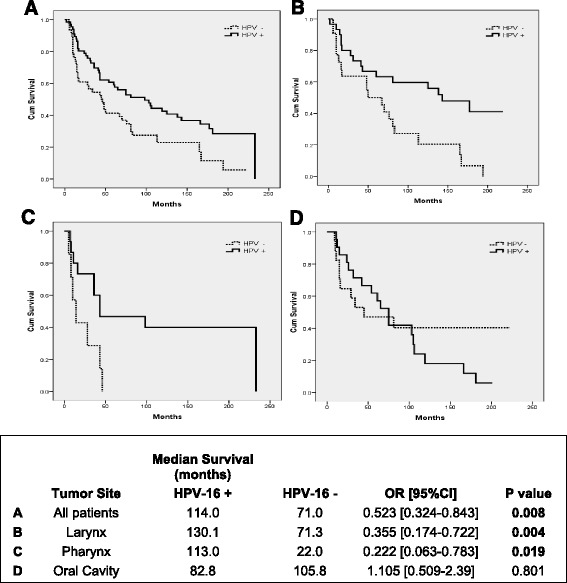
Overall survival of HNSCC patients exposed to radiation according to HPV-16 status and HNSCC sub-sites. Kaplan-Meier survival curve analysis of HNSCC patients exposed to radiotherapy (RTX) adjuvant to surgery. Patients were distributed by HPV-16 status and anatomical sub-site. **a** All RTX patients, which includes all HNSCC sub-sites and stages; **b** Larynx RTX patients; **c** Pharynx RTX patients; **d** Oral Cavity RTX patients

Recent studies have observed that HPV-16 positivity is a strong prognostic marker for OS in patients treated with primary radio-chemotherapy [30]. We compared the OS of HNSCC patients, of all sub-sites and tumor stages, which were treated with radiotherapy adjuvant to surgery. We found that HPV-16 positive patients exposed to radiotherapy after surgery had an improved survival compared to HPV-16 negative patients (Fig. 4a). Furthermore, when we sub-divide HNSCC patients by tumor site and radiotherapy treatment, HPV-16 positive LSCC and PSCC patients showed a better OS than HPV-16 negative patients (Fig. 4b, 4c). In contrast, OSCC patients, treated with adjuvant radiotherapy, do not show difference in OS in the presence or absence of HPV-16 (Fig. 4d).

**Fig. 5:**
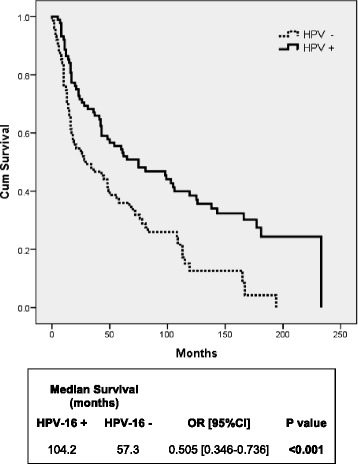
Overall survival of HNSCC patients with heavy smoking history according to HPV-16 status. Kaplan-Meier survival curve analysis of HPV-16 positive versus HPV-16 negative HNSCC patients with a history of heavy smoking

**Fig. 6:**
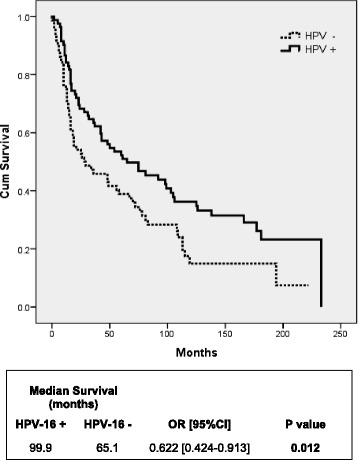
Overall survival of HNSCC patients with heavy alcohol consumption history according to HPV-16 status. Kaplan-Meier survival curve analysis of HPV-16 positive versus HPV-16 negative HNSCC patients with a history of heavy alcohol consumption

Since our study cohort was mostly composed of heavy smokers (88 %), we evaluated if HPV-16 status affects the OS of a HNSCC smoker population. As shown on Fig. 5, HPV-16 positive heavy smokers had a better OS (104.2 months) compared to HPV-16 negative heavy smokers (57.3 months) (*P* = <0.001). Likewise, the OS of HPV-16 positive patients with a history of heavy alcohol consumption was better (99.9 months) than HPV-16 negative patients (65.1 months) (*P* = 0.012) (Fig. 6).

## Discussion

Our study shows that HPV-16 positivity modifies the OS of HNSCC patients in two anatomical sub-sites, PSCC and LSCC, but not in OSCC (Fig. 2). Previous studies have shown that HPV-16 infection in LSCC and PSCC increased the survival of HNSCC patients [11, 31]. In contrast, a recent study shows that HPV-16 infection in OSCC does not cause an increase in survival, supporting our findings [32].

The key mechanism for which HPV-16 infection gives a better prognosis to HNSCC patients is still unknown. However, it has been proposed that HPV-16 positive cells have an increased sensitivity to cancer therapies, a slower cellular growth rate, an enhanced immune response towards the virus, or a combination of these factors [33]. Additionally, it has been shown that HPV-16 positive cells have fewer DNA copy number alterations, less genome-wide hypomethylation, less *TP53* mutations, and lower expression of *EGFR* [34]. Because of those differences, two HNSCC carcinogenesis models have being proposed. The first model suggests that progression of HNSCC, not infected by HPV-16, may be due to the amplification or loss of large parts of chromosome arms 3p, 9p, 11p and 17p [35–37]. In contrast, the second model of HNSCC carcinogenesis suggests that tumors infected with HPV-16 have a lower level of chromosomal loss at these regions, which may be the cause for a better survival in these patients [37].

It has been proposed that HPV-16 positivity in HNSCC produces distinct tumor sub-site differences when exposure risks are combined, suggesting that different molecular pathways are involved [38]. When we evaluated HPV-16 presence in each HNSCC anatomic sub-site, we demonstrated that HPV-16 status has a unique impact in the patient’s survival. HPV-16 positive LSCC and PSCC patients have an improved survival, in contrast to OSCC patients which did not show an improved survival. This difference may arise due to smoking and drinking habits of our study population. Our study cohort is composed of heavy smokers (88.1 %) and heavy drinkers (83.2 %). It has been proposed that HNSCC carcinogenesis in HPV-16 positive tumors, with a history of heavy smoking, may arise upon HPV-16 infection in pre-neoplastic tissue already having a number of genetic alterations, for instance, *p53* mutations or an increase in *EGFR* expression [39]. If such alterations are acquired prior to HPV-16 infection, it may impart some of the molecular characteristics of HPV-16 negative tumors, thus resulting in poor outcome and prognosis [40]. Also, it has been suggested that tobacco, alcohol and HPV-16 are independent risk factors for HNSCC, producing distinct tumor groups with different prognosis and guide of treatment [41].

HPV-16 status is an important factor for establishing the prognosis and treatment of HNSCC. Our results showed that HPV-16 positive patients had a better response to radiotherapy when compared to HPV-16 negative patients. This increase in radiosensitivity could be mediated through wild–type p53-mediated apoptosis in HPV-16 positive cells and a lower chromosomal loss [42].

The primary limitations of this study are the small number of early stages (I and II) HNSCC samples, and a small group of PSCC’s in our study cohort. These limitations may explain why we did not observe a statistical difference in early stage tumors by HPV-16 status. Our HNSCC patient population is composed mostly of late stage tumors (III, IV), predominantly from LSCC and OSCC. This issue occurs because our head and neck cancer service is attached to a supraterciary level hospital care center which is responsible for the management of complex cancer cases for the whole island of Puerto Rico.

## Conclusions

In this study we showed that, HPV-16 presence, in HNSCC tumors, causes an increase in OS and increases radiosensitivity of tumor cells. Interestingly, we have shown that HPV-16 is present, not only in OPSCC as previously described, but it was also detected in LSCC and OSCC. Although the TNM classification has been effective for prognostication of HNSCC, HPV-16 detection may serve as a potential biomarker, in combination with the TNM, to better establish the prognosis of LSCC and PSCC and guide treatment. Future work should be directed to understand how HPV-16 affects HNSCC carcinogenesis, and how its infection modifies the disease progression increasing the OS in these patients.

## Abbreviations

HNSCC, head and neck squamous cell carcinoma; HPV, human papillomavirus; IHC, immunohistochemistry; LSCC, larynx squamous cell carcinoma; OPSCC, oropharynx squamous cell carcinoma; OS, overall survival; OSCC, oral cavity squamous cell carcinoma; PSCC, pharynx squamous cell carcinoma; qPCR, quantitative real-time polymerase chain reaction; RTX, radiotherapy; TNM, TNM classification of malignant tumors
